# Expression of a small (p)ppGpp synthetase, YwaC, in the (p)ppGpp^0^ mutant of *Bacillus subtilis* triggers YvyD-dependent dimerization of ribosome

**DOI:** 10.1002/mbo3.16

**Published:** 2012-06

**Authors:** Kazumi Tagami, Hideaki Nanamiya, Yuka Kazo, Marie Maehashi, Shota Suzuki, Eri Namba, Masahiro Hoshiya, Ryo Hanai, Yuzuru Tozawa, Takuya Morimoto, Naotake Ogasawara, Yasushi Kageyama, Katsutoshi Ara, Katsuya Ozaki, Masaki Yoshida, Haruko Kuroiwa, Tsuneyoshi Kuroiwa, Yoshiaki Ohashi, Fujio Kawamura

**Affiliations:** 1 Department of Life Science, College of Science, Rikkyo UniversityToshima-ku Nishi-ikebukuro 3-34-1, Tokyo, 171-8501 Japan; 2 Cell-Free Science and Technology Research Center, Ehime UniversityBunkyo-cho, Matsuyama 790-8577 Japan; 3 Research Center for Life Science, College of Science, Rikkyo UniversityToshima-ku Nishi-ikebukuro 3-34-1, Tokyo, 171-8501 Japan; 4 Biological Science Laboratories, Kao Corporation2606 Akabane, Ichikai, Haga, Tochigi 321-3497 Japan; 5 Graduate School of Information Science, Nara Institute of Science and TechnologyIkoma, Nara 630-0101 Japan; 6 Research Information Center for Extremophile, College of Science, Rikkyo UniversityToshima-ku Nishi-ikebukuro 3-34-1, Tokyo, 171-8501 Japan; 7 Human Metabolome Technologies, Inc.246-2 Mizukami, Kakuganji, Tsuruoka, Yamagata 997-0052 Japan

**Keywords:** *Bacillus subtilis*, (p)ppGpp, (p)ppGpp synthetase, ribosome dimerization, stringent response, YvyD

## Abstract

To elucidate the biological functions of small (p)ppGpp synthetases YjbM and YwaC of *Bacillus subtilis*, we constructed RIK1059 and RIK1066 strains carrying isopropyl-β-D-thiogalactopyranoside (IPTG) inducible *yjbM* and *ywaC* genes, respectively, in the Δ*relA* Δ*yjbM* Δ*ywaC* triple mutant background. While the uninduced and IPTG-induced RIK1059 cells grew similarly in LB medium, the growth of RIK1066 cells was arrested following the addition of IPTG during the early exponential growth phase. Induction of YwaC expression by IPTG also severely decreased the intracellular GTP level and drastically altered the transcriptional profile in RIK1066 cells. Sucrose density gradient centrifugation analysis of the ribosomal fractions prepared from the IPTG-induced RIK1066 cells revealed three peaks corresponding to 30S, 50S, and 70S ribosome particles, and also an extra peak. Electron microscope studies revealed that the extra peak fraction contained dimers of 70S ribosomes, which were similar to the *Escherichia coli* 100S ribosomes. Proteomic analysis revealed that the 70S dimer contained an extra protein, YvyD, in addition to those found in the 70S ribosome. Accordingly, strain resulting from the disruption of the *yvyD* gene in the RIK1066 cells was unable to form 70S dimers following IPTG induction, indicating that YvyD is required for the formation of these dimers in *B. subtilis*.

## Introduction

In many bacteria, the stringent response is one of the global regulatory systems that is triggered by growth-limiting conditions such as amino acid starvation (Cashel et al. 1996; Magnusson et al. 2005). When a stringent response is induced, guanosine 5′-diphosphate 3′-diphosphate (ppGpp) and guanosine 5′-triphosphate 3′-diphosphate (pppGpp), which are generally referred to as (p)ppGpp, are transiently accumulated in cells and in turn control several cellular processes, including transcription, translation, nucleotide metabolism, and DNA replication (Hou et al. 1999; Artsimovitch et al. 2004; Milon et al. 2006; Wang et al. 2007). In *Escherichia coli,* cellular accumulation of (p)ppGpp is governed by two homologous enzymes, RelA and SpoT. RelA is a ribosome-associated (p)ppGpp synthetase that catalyzes the transfer of a pyrophosphoryl group from ATP to the 3′-hydroxyl group of GTP. Owing to the fact that RelA is activated by the arrival of an uncharged tRNA at the ribosome, it acts as a sensor of amino acid starvation (Wendrich et al. 2002). SpoT is a bifunctional enzyme that functions both as a (p)ppGpp synthetase as well as a hydrolase, and it regulates the level of (p)ppGpp in response to the limited availability of carbon sources, fatty acids or iron (Xiao et al. 1991; Seyfzadeh et al. 1993; Vinella et al. 2005; Battesti and Bouveret 2006). In contrast to *E. coli*, most Gram-positive bacteria were thought to possess only one RelA-SpoT homologue, designated as Rel, which has both (p)ppGpp synthesis and hydrolase activities (Wendrich and Marahiel 1997; Mittenhuber 2001). Recently, we have identified two small RelA homologues in *Bacillus subtilis*, namely YjbM and YwaC, that are capable of synthesizing (p)ppGpp (Nanamiya et al. 2008). Putative homologues of RelA, YjbM, and YwaC were also found in *Streptococcus mutans* and many other Gram-positive bacteria (Lemos et al. 2007; Nanamiya et al. 2008), which suggests that the intracellular (p)ppGpp levels in these bacteria are also controlled by these three enzymes. In addition, a novel ppGpp synthetase gene, *relV*, whose product has only weak homology to YjbM/YwaC, was found recently in a γ-proteobacterium, *Vibrio cholerae* (Das et al. 2009). Taken together, these results suggest the existence of a diverse population of (p)ppGpp synthetases in bacteria.

Recently, it was shown that the transcription of rRNA operons from their P1 promoters in *B. subtilis* was drastically reduced in the *relA* mutant, but the transcription was restored almost completely in the Δ*relA* Δ*yjbM* Δ*ywaC* triple null mutant (Natori et al. 2009). It was also observed that the intracellular GTP concentration was reduced in the *relA* mutant, but was restored in the Δ*relA* Δ*yjbM* Δ*ywaC* triple mutant. These results suggest that YjbM and/or YwaC continuously synthesize (p)ppGpp at the basal level, which causes a decrease in the intracellular GTP level (Nanamiya et al. 2008). However, the exact contribution of YjbM and/or YwaC to (p)ppGpp production at the basal level, as well as the biological functions of these enzymes, remains unclear. To address these questions, we have developed unique strains in which either YjbM or YwaC could be expressed as the only (p)ppGpp synthetase, and used these strains to examine the effects of their individual expression on cell proliferation and ribosome formation. We presented evidence that YwaC induces the transcription of *yvyD* via a sporulation-specific sigma factor, σ^H^. In addition, we showed that the resulting YvyD protein is essential for the dimerization of 70S ribosomes.

## Results

### Growth characteristics of Δ*relA* Δ*yjbM* Δ*ywaC* triple mutant carrying inducible *yjbM* or *ywaC* gene

**Figure 1:**
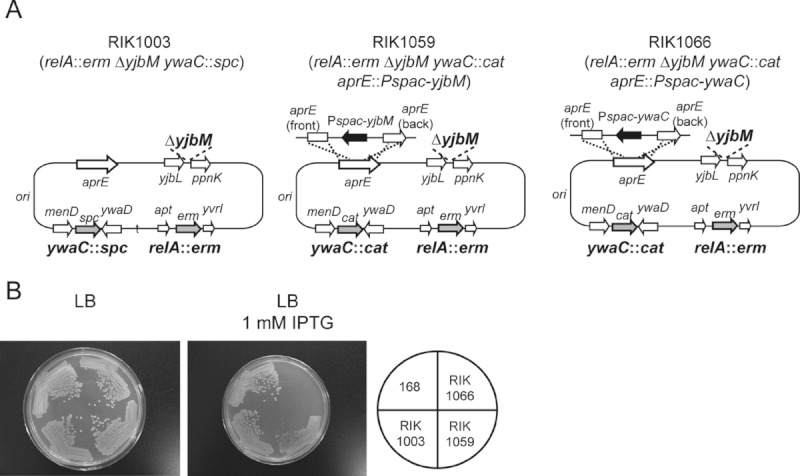
Growth characteristics of the triple mutants carrying the IPTG-inducible *yjbM* or *ywaC* genes. (A) Schematic representations of the chromosome structures of the strains RIK1003 (*relA*::*erm* Δ*yjbM ywaC*::*spc*), RIK1059 (*aprE*::P*spac*-*yjbM relA*::*erm* Δ*yjbM ywaC*::*cat*), and RIK1066 (*aprE*::P*spac*-*ywaC relA*::*erm* Δ*yjbM ywaC*::*cat*). (B) Growth of wild-type (168), RIK1003, RIK1059, and RIK1066 cells on LB plates with or without 1 mM IPTG.

To determine the biological functions of YjbM and YwaC, the *yjbM* and *ywaC* genes were cloned individually under the control of an isopropyl-β-D-thiogalactopyranoside (IPTG) inducible P*spac* promoter and each clone was then separately inserted at the *aprE* site of the chromosome of a Δ*relA* Δ*yjbM* Δ*ywaC* triple null mutant strain (Fig. 1A). This resulted in strains RIK1059 (*aprE*::P*spac*-*yjbM* Δ*relA* Δ*yjbM* Δ*ywaC*) and RIK1066 (*aprE*::P*spac*-*ywaC* Δ*relA* Δ*yjbM* Δ*ywaC*), which could be used to express YjbM and YwaC, respectively, after induction with IPTG.

**Figure 2:**
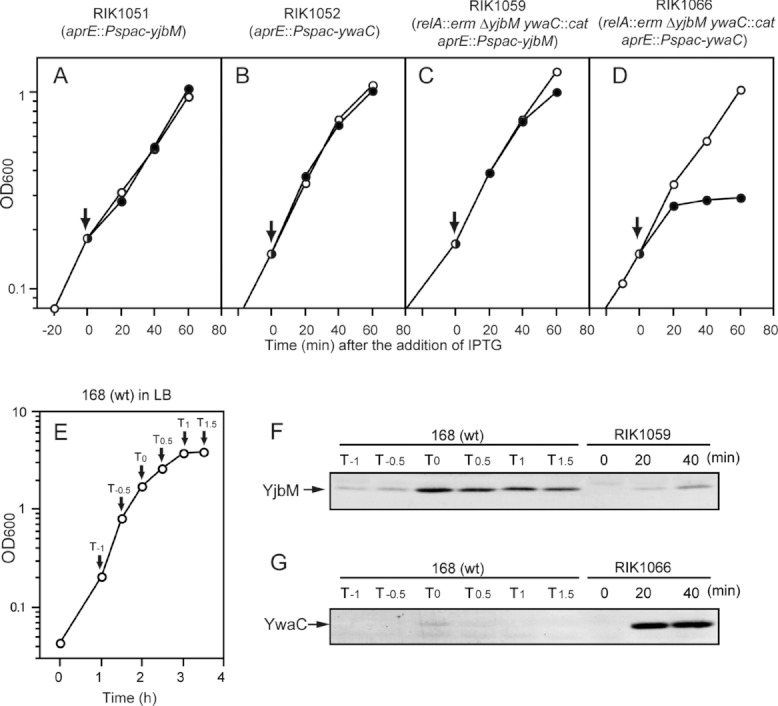
Growth profiles of (A) RIK1051 (*aprE*::P*spac*-*yjbM*), (B) RIK1052 (*aprE*::P*spac*-*ywaC*), (C) RIK1059 (*aprE*::P*spac*-*yjbM relA*::*erm* Δ*yjbM ywaC*::*cat*), and (D) RIK1066 (*aprE*::P*spac*-*ywaC relA*::*erm* Δ*yjbM ywaC*::*cat*) in LB medium. Cells were inoculated into LB medium and grown at 37°C with shaking. When the optical density at 600 nm (OD_600_) reached ca. 0.2 (at times indicated by the arrows), IPTG was added to the culture to a final concentration of 1 mM and cell growth was monitored by measuring OD_600_ of the culture: cells with (?) or without (○) IPTG. (E) Growth curve of a wild-type strain (168) of *Bacillus subtilis*. Cells were incubated in LB medium at 37°C with shaking and were collected at the indicated times after inoculation for measurement of OD_600._ Cells were also collected at the times indicated by dotted allows for Western blot analyses, results of which are shown in panels (F) and (G). (F and G) Intracellular levels of (F) YjbM and (G) YwaC in 168 (wild-type) cells during the indicated times of the growth phase as shown in (E); intracellular levels of YjbM in (F) RIK1059 and that of YwaC in (G) RIK1066 cells before (time 0) and after the addition of IPTG are also shown. Crude cell extracts were subjected to Western blot analyses using (F) anti-YjbM and (G) anti-YwaC antibodies, respectively, as described in the Section Experimental procedures.

In contrast to the RIK1059 strain, the RIK1066 strain, which carried the IPTG-inducible *ywaC* gene in the triple mutant background, did not produce any visible colony when it was incubated on LB agar plates supplemented with 1 mM IPTG at 37°C (Fig. 1B), which suggests that the expression of YwaC arrests the growth of cells carrying the Δ*relA* Δ*yjbM* Δ*ywaC* triple null mutations. To confirm whether the expression of YwaC indeed affects the cell proliferation, we first created RIK1051 and RIK1052 strains that carried the IPTG-inducible *yjbM* gene and IPTG-inducible *ywaC* gene, respectively, in the wild-type background. We then grew RIK1051, RIK1052, RIK1059, and RIK1066 cells in LB medium and induced them with 1 mM IPTG at the early growth phase. As shown in Figure 2A and B, addition of IPTG did not affect the growth rates of RIK1051 and RIK1052, the wild-type cells carrying the inducible *yjbM* or *ywaC* gene, respectively. The growth rate of the RIK1059 cells (inducible *yjbM* gene carrying triple mutant cells) also remained unchanged after IPTG addition (Fig. 2C). In contrast, growth rate of the RIK1066 cells (inducible *ywaC* gene carrying triple mutant cells) decreased 20 min after the addition of IPTG, following which they almost ceased to grow (Fig. 2D). It is clear from these results that inducing the expression of *ywaC* gene in the Δ*relA* Δ*yjbM* Δ*ywaC* triple mutant prevents the growth of these cells.

In order to confirm the expression of YjbM or YwaC in the Δ*relA* Δ*yjbM* Δ*ywaC* triple mutant, as well as in the wild-type cells grown in LB medium, Western blot analyses were performed using anti-YjbM or anti-YwaC antibodies. In the wild-type cells, expression of the YjbM protein was detected all through its growth in LB medium at 37°C, and the expression level reached a maximum at the transient state of growth (T_0_; Fig. 2E and F). These results were somewhat different from the previous study in which it was shown that the transcription of *yjbM* was constitutive from the early exponential phase through the early-stationary growth phase of the wild-type cells grown in LB medium at 37°C (Nanamiya et al. 2008). It is thus possible that the intracellular level of YjbM during the early exponential growth phase might be regulated by an unknown posttranscriptional regulatory mechanism. The intracellular level of YjbM in the IPTG-induced RIK1059 cells (Δ*relA* Δ*yjbM* Δ*ywaC* triple mutant harboring IPTG-inducible *yjbM*) was similar to those of the wild-type cells during the early exponential growth phase (Fig. 2F). Given that the expression level of YjbM in RIK1059 was lower than that observed at the transient growth state of the wild-type cells, the expressed YjbM in the triple mutant may not be able to exert full effect on cell proliferation. The expressed YwaC, however, was only faintly detected at the transition growth state of the wild-type cells (Fig. 2E and G). This result was generally consistent with previous transcription analyses of *ywaC* (Nanamiya et al. 2008). On the other hand, the intracellular level of YwaC in RIK1066 cells synthesized after the IPTG induction was much higher than that in the wild-type cells (Fig. 2G). Therefore, it is possible that the high expression level of YwaC in the triple mutant severely affected the proliferation of RIK1066 cells.

### Isolation and characterization of the suppressor mutations that overcome the growth arrest as a result of induced expression of *ywaC* in RIK1066

The growth arrest caused by the induced expression of *ywaC* in RIK1066 cells was also observed in RIK1068 (*aprE*::P*spac*-*ywaC* Δ*relA* Δ*yjbM*), RIK1098 (*aprE*::P*spac*-*ywaC* Δ*relA* Δ*ywaC*), and RIK1054 (Δ*relA aprE*::P*spac*-*ywaC*) cells (Fig. S1). Therefore, it is most likely that the expression of YwaC in the absence of RelA caused growth arrest in these cells. In order to understand the underlying mechanism, we isolated suppressor mutants of RIK1066 (*aprE*::P*spac*-*ywaC* Δ*relA* Δ*yjbM* Δ*ywaC*) cells from LB agar plates supplemented with 1 mM IPTG (Table S1). All suppressor mutants examined were found to have mutations in the *ywaC* gene, including a nonsense mutation and various deletion mutations (Table S1). Two of these suppressor mutants, RIK1371 (*aprE*::P*spac*-*ywaCL176F* Δ*relA* Δ*yjbM* Δ*ywaC*) and RIK1375 (*aprE*::P*spac*-*ywaCD87G* Δ*relA* Δ*yjbM* Δ*ywaC*), were found to contain a single-base substitution in the coding region of *ywaC* gene (Table S1). As expected, growths of these two suppressor mutants, unlike their parent strain RIK1066, were not arrested by the addition of 1 mM IPTG (Fig. S1).

RIK1375 strain carried a point mutation (A260G) in the *ywaC* open reading frame of the P*spac-ywaC* gene (Table S1), resulting in substitution of the Asp87 residue of YwaC protein to a Gly residue. This Asp residue is conserved among the RelA/SpoT family of proteins and is thought to be involved in the binding of Mg^2+^ in the presence of ATP (Hogg et al. 2004; Nanamiya et al. 2008). Replacement of the Asp residue at this position with a Gly residue has shown to ablate lost the (p)ppGpp synthesizing ability of the RelA/SpoT proteins (Hogg et al. 2004; Nanamiya et al. 2008). Therefore, it is most likely that the expression of YwaCD87G protein, which lacks the ability to synthesize (p)ppGpp (or other guanine nucleotides as described below), in the Δ*relA* Δ*yjbM* Δ*ywaC* triple mutant cells would not affect their growth.

### Effects of expression of *ywaC* gene on the intracellular concentrations of various guanine nucleotides in the Δ*relA* Δ*yjbM* Δ*ywaC* triple mutant

**Figure 3:**
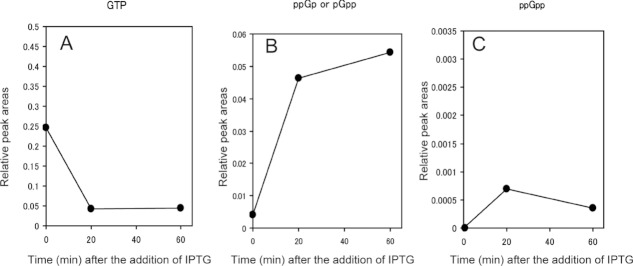
Intracellular concentration of (A) GTP, (B) pGp/ppGp, and (C) ppGpp in RIK1066 (*aprE*::P*spac*-*ywaC relA*::*erm* Δ*yjbM ywaC*::*cat*) cells. Cells were grown in LB medium at 37°C with shaking and harvested at the times indicated before (time 0) or after the addition of IPTG. Crude cell extracts were subjected to capillary electrophoresis time-of-flight mass spectrometry (CE-TOFMS) analysis as described in the Section Experimental procedures.

Among all other small ppGpp synthetases in *B. subtilis*, only RelA contains a HD domain, which defines a superfamily of metal-dependent phosphohydrolase (Aravind and Koonin 1998; Nanamiya et al. 2008). It is thus possible that the hydrolase activity of RelA plays a pivotal role in maintaining the normal growth after the induction of the *ywaC* gene, because the expressed YwaC was able to convert GTP or GDP into (p)ppGpp or other unknown guanine nucleotides. Therefore, we next determined the effects of expressing the *ywaC* gene on the intracellular concentrations of various guanine nucleotides in the Δ*relA* Δ*yjbM* Δ*ywaC* triple mutant by using capillary electrophoresis time-of-flight mass spectrometry (CE-TOFMS). Inducing the expression of *ywaC* gene by IPTG drastically reduced the intracellular concentration of GTP in RIK1066 cells (Fig. 3A). Although we detected (p)ppGpp in the cell extracts of IPTG-induced RIK1066 cells (Fig. 3C), the amount of (p)ppGpp detected in these cells was extremely low compared to the levels usually found after amino acid deprivation. In fact, determination of the intracellular levels of (p)ppGpp by HPLC, as described previously (Nanamiya et al. 2008), in the cell extracts of IPTG-induced Δ*relA* Δ*yjbM* Δ*ywaC* triple mutant cells expressing YwaC was unsuccessful (data not shown). In contrast, the intracellular levels of guanine nucleotide derivatives pGpp and/or ppGpp, which were indistinguishable by this analysis, were more than the intracellular level of (p)ppGpp (Fig. 3B and C). These changes in the intracellular levels of various guanine nucleotides might account for the growth arrest of the IPTG-induced RIK1066 cells that were expressing YwaC in the Δ*relA* Δ*yjbM* Δ*ywaC* triple mutant background.

### Effects of the expression of *ywaC* on transcriptome

**Figure 4:**
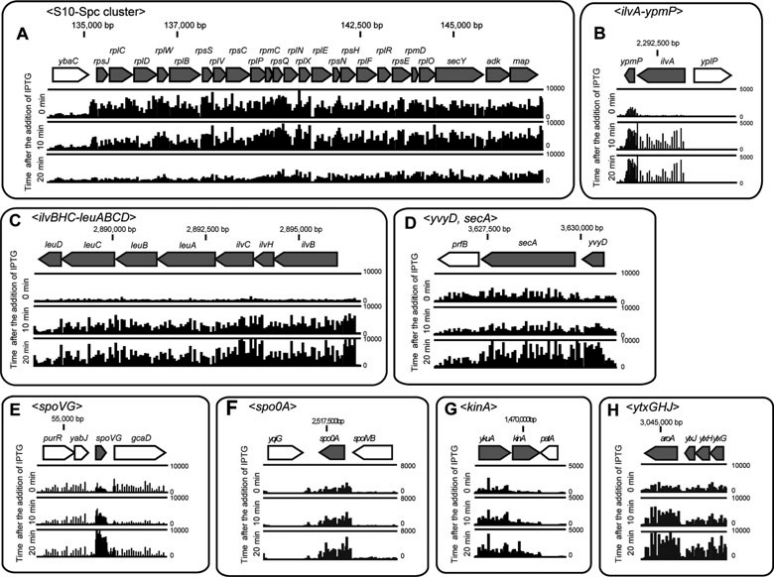
Effects of inducing expression of the *ywaC* gene on the transcriptome of the Δ*relA* Δ*yjbM* Δ*ywaC* triple mutant. RIK1066 cells were grown in LB medium at 37°C with shaking. Cells were harvested at the times indicated before (time 0) or after the addition of IPTG. Total RNAs extracted from the harvested cells were subjected to transcriptome analysis using the high-density tiling chip as described in the Section Experimental procedures. The transcriptional profiles of (A) S10-Spc cluster, (B) *ilvA-ypmA*, (C) *ilvBHC-leuABCD*, (D) *yvyD-secA,* (E) *spoVG,* (F) *spo0Aps,* (G) *kina,* and (H) *ytxGHJ* were shown. Transcriptional signal for each probe is shown using a vertical bar at the appropriate genomic coordinate. Arrangements of genes around selected gene(s), indicated by gray arrowhead bars, are schematically shown on top of the vertical bars.

To obtain further information on the effect of YwaC expression, transcriptome analysis of RIK1066 cells was carried out by using high-density tiling chips as described in the Section Experimental procedures. Among 4176 genes found in *B. subtilis* genome (Kunst et al. 1997), we actually obtained transcriptional profiles of 2427 genes as significant signals (Table S2). These profiles were then processed by k-means algorithms and divided into nine clusters (Table S3; Fig. S2). Transcription levels of genes found in the clusters 1, 5, 6, 9 (789 genes in total) were reduced after inducing the expression of the *ywaC* gene in RIK1066 cells with IPTG (Table S3; Fig. S2). The S10-Spc cluster, in which many of the genes encoding ribosomal proteins are located, was one among these gene clusters (Fig. 4A). Transcription of the genes in clusters 2 and 4 (366 genes in total), on the other hand, remarkably increased after inducing the expression of the *ywaC* gene (Table S3; Fig. S2). Among these genes were the genes for the branched chain amino acid (*ilv-leu*) synthesis (Fig. 4B and C) as well as the genes encoding the alpha-acetolactate synthase (AlsS), alpha-acetolactate decarboxylase (AlsD), and pyruvate carboxylase (PycA) (Table S3) (Diesterhaft and Freese 1973; Renna et al. 1993). It was reported earlier that the intracellular GTP level in *B. subtilis* decreased when they were treated with decoynine, a specific inhibitor for GMP synthesis (Mitani et al. 1977; Lopez et al. 1981; Inaoka et al. 2003, Tojo et al. 2010). In a recent study, transcriptome analysis carried out under this condition showed that the expressions of *ilv-leu*, *pycA,* and *alsSD* genes increased, whereas the expressions of various genes encoding the ribosomal proteins decreased (Tojo et al. 2010). Results shown in this study (Fig. 4; Tables S2 and S3) are consistent with these previous observations. Taken together, our results strongly suggest that a decrease in the intracellular GTP level causes the dramatic changes in the transcriptional profile of the RIK1066 cells.

### Effects of the expression of *ywaC* on ribosome formation

**Figure 5:**
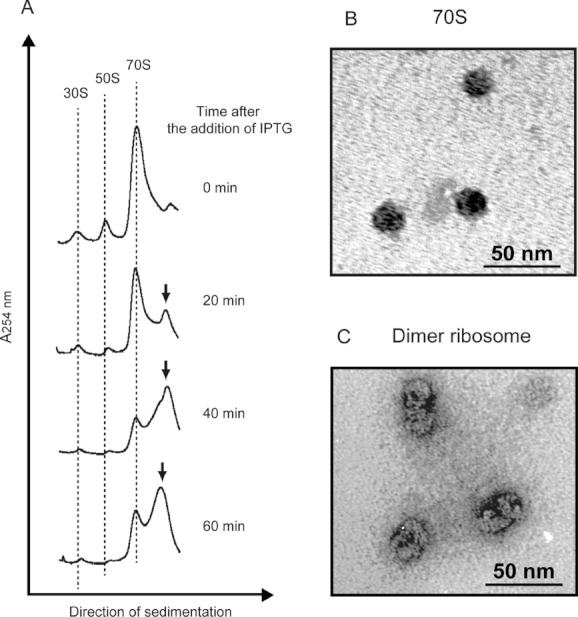
(A) Effects of inducing the expression of *ywaC* gene on ribosome formation in RIK1066 cells. Growth of RIK1066 cells, IPTG treatment, and harvesting of cells were carried out exactly as described in the legend of Figure 4. Crude cell extracts were prepared from the harvested cells. Each cell extract was then sedimented through a 10–40% sucrose gradient as described in the Section Experimental procedures and the obtained sedimentation profiles are shown in this panel. The arrow indicates the additional peak. (B and C) Electron micrographs of the fractions corresponding to (B) 70S peak and (C) additional peak. RIK1066 cells were grown in LB medium at 37°C with shaking, IPTG was added at OD_600_ = 0.2 and cells were harvested 60 min after the addition of IPTG. The peak fractions corresponding to 70S and additional peak were separately pooled and each fraction was further purified as described in the Section Experimental procedures.

Next, we determined the sedimentation profiles of ribosomes by sucrose density gradient centrifugation analysis of the cell extracts of RIK1066 cells before and after IPTG induction. As shown in Figure 5A, the sedimentation profile of the cell extracts prepared from the induced RIK1066 cells showed an extra peak in addition to the peaks of 30S, 50S, and 70S ribosomes. The height of this extra peak increased with the time of incubation with IPTG (Fig. 5A). It was previously shown that during the stationary growth phase the 70S ribosome of *E. coli* dimerizes to form the so-called 100S ribosome (Wada et al. 1990). Given that the additional peak shown in Figure 5A was found at a similar S-value to that of the 100S ribosome of *E. coli*, it is possible that the additional peak fraction contains dimers of 70S ribosomes. We have also found that polysomes often sediment on the sucrose density gradient at an S-value similar to that of the additional peak. To examine the possibility that the additional fraction might contain polysomes, crude extracts were subjected to sedimentation analysis after 60 min of incubation at 37°C. If the peak fraction indeed contained active ribosome particles, such as polysomes consisting of several 70S ribosomes bound to mRNAs, then we would expect the peak intensity to decrease as a result of dissociation of 70S particles from the mRNAs. As shown in Fig. S3, relative intensities of the peaks corresponding to polysomes of RIK1052 (*aprE*::P*spac*-*ywaC*) apparently decreased after preincubation at 37°C. On the other hand, intensities of the additional peaks found in the extracts of the induced RIK1066 cells remained almost similar (Fig. S3). These results strongly suggest that the additional peak contains 70S ribosomes, which was qualitatively different from the active 70S particles, such as polysomes.

The additional peak fraction was next analyzed by electron microscopy. We found dimers of the 70S ribosomes in the pooled fraction (Fig. 5C). In contrast to the100S ribosome of *E. coli*, where two 70S ribosomes contact each other via a face-to-face interaction with the two 30S subunits (Wada 1998), we were unable to spot any significant structural differences among the 70S dimers (Fig. 5B and C). Nevertheless, these results provided the first experimental evidence of dimerization of the 70S ribosome in *B. subtilis* cells, although information on the detailed structure of these 70S dimers remained elusive.

### YvyD is involved in the dimerization of 70S ribosome

**Figure 6:**
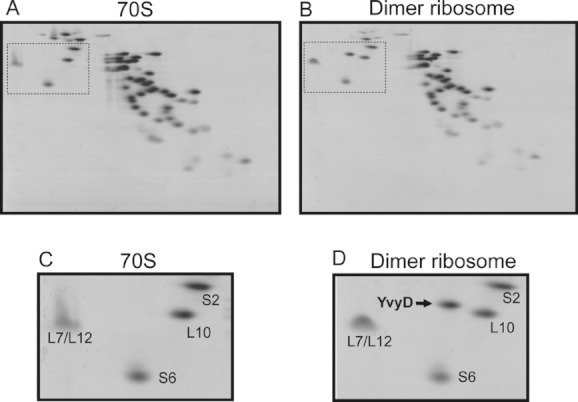
RFHR 2D PAGE of (A) 70S ribosome and (B) 70S dimer fractions. RIK1066 cells were grown and induced with IPTG for 60 min as described in the legend of Figure 2. The 70S and 70S dimer peaks were fractionated by sucrose density gradient from the cell extract and were further purified. Subsequently, they were subjected to 2D-PAGE analysis as described in the Section Experimental procedures. Protein spots from the boxed areas of the stained 2D gels shown in (A) and (B) are magnified in (C) and (D), respectively. Each one of these spots were extracted and identified by mass spectrometry.

**Figure 7:**
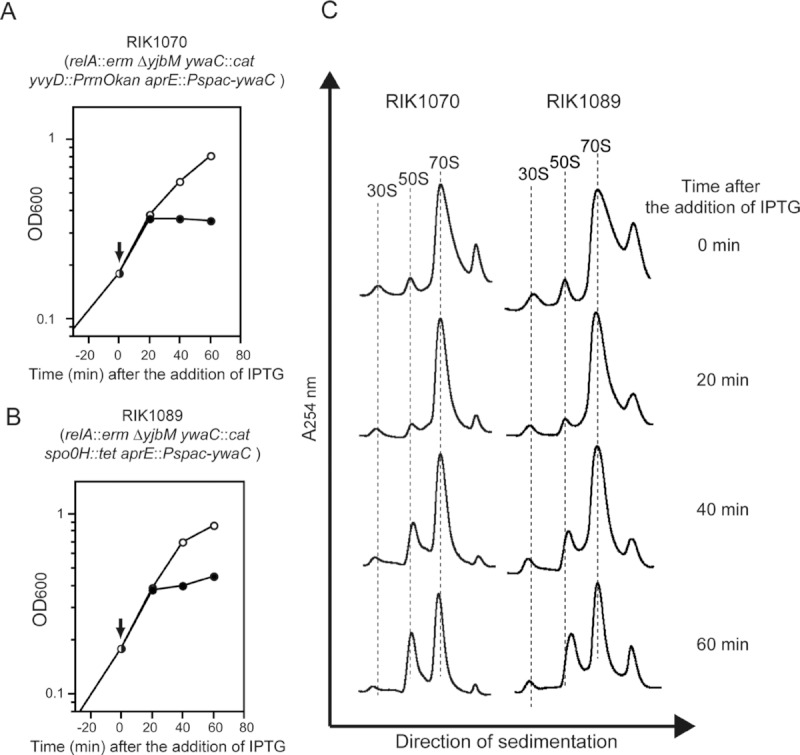
Effects of disruption of *yvyD* and *spo0H* genes on cell proliferation and ribosome formation. (A and B) Cell proliferation. Growth profiles of (A) *yvyD* gene disrupted strain RIK1070 (*aprE*::P*spac*-*ywaC relA*::*erm* Δ*yjbM ywaC*::*cat yvyD*::P*rrnOkan*) and (B) *spo0H* gene disrupted strain RIK1089 (*aprE*::P*spac*-*ywaC relA*::*erm* Δ*yjbM ywaC*::*cat spo0H*::*tet*) in LB medium. Cells were inoculated in LB medium and grown at 37°C with shaking. When the OD_600_ reached ca. 0.2 (indicated by arrow), 1 mM IPTG (final concentration) was added to the culture and the OD_600_ of the culture was monitored: (?) cells treated with IPTG (○) untreated cells (control). (C) Ribosome formation. Sedimentation profiles of sucrose density gradients of IPTG-induced and uninduced cells. Crude cell extracts were prepared from IPTG-induced and uninduced RIK1070 and RIK1089 cells and the cell extracts were then sedimented through a 10–40% sucrose gradient as described in the Section Experimental procedures.

It has been shown in *E. coli* that the formation of 100S ribosome requires ribosome-associated proteins such as ribosome modulation factor [RMF] (Wada et al. 1990). However, no homologue of RMF gene was found in the genome of *B. subtilis* (Kunst et al. 1997). Thus, it is possible that in *B. subtilis* dimerization of the 70S ribosome is controlled by factors different from those in *E. coli*, and they might have distinct modes of action. To explore this possibility, the peak fraction containing the ribosome dimer was prepared from the IPTG-induced cells, and subsequently the ribosomal proteins were separated by radical free and highly reducing (RFHR) two-dimensional (2D) gel electrophoresis. As shown in Figure 6, an additional protein spot was found in the dimer ribosome fraction and this protein was not detected in the 70S ribosome fraction. Matrix-assisted laser desorption/ionization time-of-flight (MALDI-TOF) analysis identified this spot as YvyD protein, which was also identified in a previous study involving proteomic analysis of ribosomal proteins (Nanamiya et al. 2004). Densitometric scanning revealed that the relative staining intensity of the YvyD spot was about 80% of the relative staining intensities of the neighboring spots L10 and S2. Therefore, the molecular ratio between YvyD and 70S ribosome is most likely 1:1. The presence of YvyD in the ribosome dimer fraction suggests that this protein might be involved in the dimerization of 70S ribosome. To test this idea, the *yvyD* gene in RIK1066 strain was disrupted. The resultant mutant strain, RIK1070 (*aprE*::P*spac*-*ywaC* Δ*relA* Δ*yjbM* Δ*ywaC* Δ*yvyD)*, was incubated in LB medium at 37°C with shaking and IPTG was added to a final concentration of 1 mM during the early exponential growth phase (Fig. 7A). As shown in Figure 7C, sedimentation analysis of cell extracts prepared from these cells showed no significant induction of the 70S dimer containing peak, which suggests that YvyD is required for the dimerization of 70S ribosome. In addition, the area under the 70S ribosome peak was reduced and concomitantly the area under the 50S subunit peak, but not that of the 30S subunit peak, was increased with the induction time (Fig. 7C). However, growth of this strain was arrested approximately 20 min after the addition of IPTG (Fig. 7A). These results suggest that the formation of 70S ribosome dimer and the function of YvyD are independent of the growth arrest caused by the induction of *ywaC* expression in the triple mutant.

### Transcription of *yvyD* is dependent on a sporulation-specific sigma factor, σ^H^, after the induction of *ywaC*

**Figure 8:**
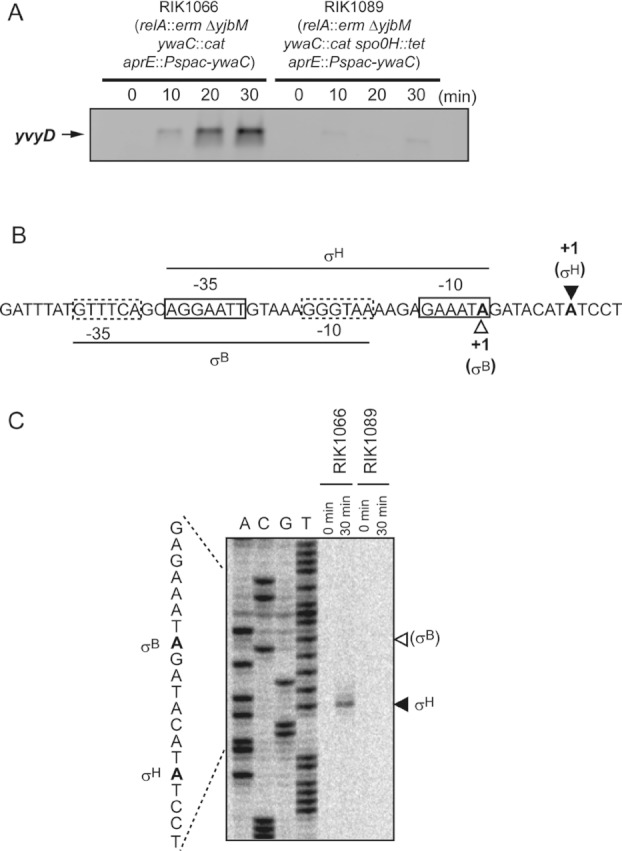
Transcription analyses of *yvyD* after inducing the expression of *ywaC*. (A) Northern blot analysis of *yvyD*. Total RNAs were extracted from the IPTG-induced and uninduced RIK1066 and RIK1089 (*aprE*::P*spac*-*ywaC relA*::*erm* Δ*yjbM ywaC*::*cat spo0H*::*tet*) cells and were used for the Northern blot analysis using a *yvyD*-specific riboprobe as described in the Section Experimental procedures. (B) DNA sequence of the *yvyD* promoter region as reported by Drzewiecki et al. (1998). –35 and –10 regions of the σ^H^- and σ^B^-dependent promoters are boxed using solid lines (σ^H^) and dotted lines (σ^B^), respectively. The two transcriptional start sites are shown in bold letters with closed (σ^H^) and open (σ^B^) arrowheads. (C) Primer extension products were generated using total RNAs isolated from IPTG-induced and uninduced RIK1066 or RIK1089 cells as described in the legend of Figure 6A. Closed and open arrowheads represent transcriptional start sites of the σ^H^- and σ^B^-dependent promoters, respectively.

As described above, the expression of YwaC in the triple mutant caused YvyD to accumulate in the fraction that contained the70S ribosome dimer. To elucidate the role of YwaC in the expression of YvyD, the transcription of *yvyD* gene in RIK1066 was analyzed first by Northern blot assay. We found that the transcription of *yvyD* was induced by the addition of IPTG to the cell culture (Fig. 8A). This result was consistent with the results of transcriptome analysis (Fig. 4E). It has been shown previously that *yvyD* is transcribed from two promoters (Fig. 8B): one is dependent on the sigma factor σ^B^, which is responsible for the transcription of genes required for the general stress response, and the other is controlled by the sigma factor σ^H^, which is required for the transcription of early sporulation genes (Varón et al. 1996; Drzewiecki et al. 1998). Results of our transcriptome analysis also revealed that the expression of the genes such as *spoVG*, *spo0A*, *kinA,* and *ytxGHJ*, whose transcription is dependent on σ^H^, was upregulated in IPTG-induced RIK1066 cells (Fig. 4E, F, G, and H; Tables S2 and S3). It is thus most likely that the induction of *yvyD* transcription in RIK1066 cells is solely dependent on the σ^H^ function. Therefore, we performed primer extension analysis to determine from which promoter *yvyD* was transcribed following the induction of *ywaC* expression in the triple mutant. As shown in Figure 8C, reverse transcripts that corresponding to the +1 position of the σ^H^-dependent promoter were indeed detected in the IPTG-induced RIK1066 cells. To examine whether the transcription of *yvyD* following the induction of *ywaC* gene was solely dependent on σ^H^, the *spo0H* gene, which encodes the σ^H^ protein, was deleted in the RIK1066 cells to create the Δ*relA* Δ*yjbM* Δ*ywaC* Δ*spo0H* quadruple mutant strain RIK1089. Total RNA purified from this quadruple mutant was then used for primer extension and Northern blot analyses. As shown in Figure 8A and C, neither the *yvyD* transcript nor the primer extended reverse transcript of *yvyD* was detected in this mutant. Furthermore, sucrose gradient ultracentrifugation analyses did not revealed any significant induction of the peak that corresponded to the 70S dimer in the Δ*relA* Δ*yjbM* Δ*ywaC* Δ*spo0H* quadruple mutant strain, even after the expression of YwaC was induced with IPTG (Fig. 7C). These results clearly indicated that *yvyD* is transcribed from the σ^H^-dependent promoter following the induction of the *ywaC* gene in the RIK1066 strain. The growth of the RIK1089 strain, which has a deletion in the *Spo0H* gene, was arrested approximately 20 min after the addition of IPTG, which was same as that was observed with the RIK1066 cells. This observation suggests that σ^H^ protein is not involved in the growth arrest of RIK1066 cells following IPTG addition.

We also found that when the mutant YwaCD87G, which lacked the (p)ppGpp synthesis activity, was expressed in the Δ*relA* Δ*yjbM* Δ*ywaC* triple mutant cells, the 70S ribosome dimer peak was not observed in the sedimentation analysis (Fig. S3). Given that YwaCD87G was actually expressed in these cells (RIK1375 cells in Fig. S4), but growth arrest was not observed (Fig. S1), it strongly suggests that the YwaC-mediated changes in the intracellular guanine nucleotide level is critical for the cell growth retardation and also suggest that YwaC itself is not directly involved in the dimerization of 70S ribosome.

### YvyD does not directly trigger dimerization of 70S ribosome

**Figure 9:**
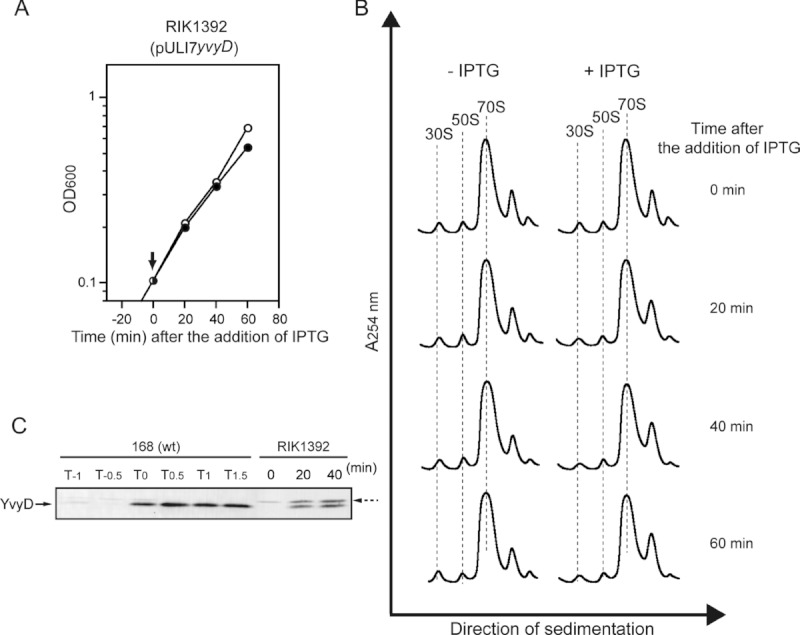
Effects of overexpression of *yvyD* on cell proliferation and ribosome formation. (A) Growth profiles of strain RIK1392 carrying pULI7*yvyD* in LB medium. Cells were inoculated in LB medium and grown at 37°C with shaking. When the OD_600_ reached ca. 0.1 (indicated by arrow), 1 mM IPTG (final concentration) was added to the culture and the OD_600_ of the culture was monitored: (?) cells treated with IPTG (○) untreated cells (control). (B) Ribosome formation. Sedimentation profiles of sucrose density gradients of IPTG-induced and uninduced cells. Crude cell extracts were prepared from IPTG-induced and uninduced RIK1392 cells and the cell extracts, which were preincubated for 60 min at 37°C, were then sedimented through a 10–40% sucrose gradient as described in the Section Experimental procedures. (C) Western blot analysis of YvyD. Crude cell extracts prepared from wild-type and RIK1392 cells grown in LB medium at 37°C (before [indicated by time 0] and after the addition of IPTG) were subjected to Western blot analysis using the anti-YvyD antibody as described in the Section Experimental procedures. Note that the additional band (shown by dotted arrow) was detected only in the cell extracts of RIK1392 cells.

From the results described above, there is little doubt that YvyD is involved in the formation of 70S dimer. However, it remains unclear whether or not YvyD directly triggers the dimerization of 70S. If YvyD protein alone had the ability to convert 70S ribosomes into 70S dimers, dimerization of 70S ribosomes would be detected due to the overexpression of YvyD in wild-type cells during exponential growth phase. To test this idea, we constructed a multicopy plasmid carrying the IPTG-inducible *yvyD* gene (pULI7yvyD) and introduced it into the wild-type strain. The intracellular level of YvyD in the wild-type cells grown in LB medium at 37°C was low and was below the detection level during the early exponential growth phase, but its expression increased after the end of exponential growth (Fig. 9C). In contrast, YvyD protein was actually synthesized in the wild-type cells carrying the pULI7yvyD plasmid after the addition of IPTG to the culture at the early exponential growth phase (Fig. 9A and C). However, the induced expression of YvyD from the plasmid during the early exponential growth phase of the wild-type cells did not have any significant effect either on the cell proliferation (Fig. 9A) or on the dimerization of 70S ribosomes (Fig. 9B). These results strongly suggested that even though YvyD is required for the 70S dimer formation, there are other mechanism(s) that triggers the formation of 70S dimers. Figure 9C also showed an additional band (indicated using a dotted arrow) that was detected only in the cell extracts of RIK1392 cells. Because this additional band was detected before the induction of YvyD expression in RIK1392 cells, it was assumed that these bands were not derived from YvyD itself.

## Discussion

The *B. subtilis* strain carrying a null mutation in the *relA* gene, whose product is involved in the synthesis and/or hydrolysis of (p)ppGpp, grows more slowly in LB medium than the wild-type strain (Nanamiya et al. 2008). This growth defect could be suppressed by introducing null mutation(s) in the *yjbM* and/or *ywaC* gene(s), both of which encode small (p)ppGpp synthetases (Nanamiya et al. 2008). Further genetic studies revealed that all suppressor mutations of *relA* null mutant contained mutations either in *yjbM* or in *ywaC*, which suggested that RelA, YjbM, and YwaC are the only (p)ppGpp synthetases in *B. subtilis* (Natori et al. 2009). These results were consistent with the results of previous genetic studies (Srivatsan et al. 2008). Detailed biological functions of YjbM and YwaC, however, still remain unknown. To investigate their functions, we constructed strains in which the expression of *yjbM* or *ywaC* could be induced by IPTG in a Δ*relA* Δ*yjbM* Δ*ywaC* triple null mutant background, and subsequently used these strains to study the effects of expression of these genes on cell proliferation as well as ribosome formation. In this study, we found that inducing the expression of the *ywaC* gene in the Δ*relA* Δ*yjbM* Δ*ywaC* triple mutant causes growth arrest, presumably due to the severe reduction in the intracellular GTP level. In addition, expression of YwaC protein in the triple mutant led to dimerization of the 70S ribosome, and showed that YvyD, whose function yet remains to be determined, was required for this dimerization.

Induction of YwaC expression in the Δ*relA* Δ*yjbM* Δ*ywaC* triple mutant arrested its growth in LB medium (Fig. 2D). This growth arrest was, however, not observed in YjbM expressing triple mutant (Fig. 2C). Additionally, mutations in the open reading frame of the inducible *ywaC* gene could bypass the growth arrest (Fig. S1). Moreover, the growth arrest caused by the induced expression of YwaC was observed only when the cells carried Δ*relA* mutation (Fig. S1). These results strongly suggest that enzymatic activity of YwaC (ability to convert GTP or GDP to (p)ppGpp or other guanine nucleotides) is critical for the growth arrest.

CE-TOFMS analysis revealed that the intracellular GTP levels were reduced in the IPTG-induced RIK1066 cells expressing YwaC (Fig. 3A). This result, in combination with the transcription results shown in Figures 3, 4 and S3, and in Tables S2 and S3, strongly suggest that the reduced level of intracellular GTP inhibited the cell growth. (p)ppGpp was actually synthesized in the RIK1066 cells expressing YwaC (Fig. 3C). Although the amounts of (p)ppGpp synthesized in RIK1066 cells following the induced expression of YwaC seemed to be very low as compared to the level of (p)ppGpp synthesized following amino acid depletion, results shown in Figure 3A suggest that YwaC, at least in part, contribute to the reduction of intracellular GTP level. CE-TOFMS analysis also revealed that guanine nucleotide derivatives, ppGp/pGpp, were detected in YwaC expressing triple mutant cells (Fig. 3B). In a previous study, we have shown that in vitro YwaC could synthesize yet uncharacterized guanine nucleotide-related compounds by transferring the γ-phosphate group of ATP to the 5′-position (γ or β position) of GTP or GDP (Nanamiya et al. 2008). Thus, it is likely that this peak would be mainly consisted of ppGp. The detailed role of these guanine-related nucleotides, however, remains unclear. One possibility is that these nucleotides, such as (p)ppGpp, inhibit GTP synthesis. The basal level of (p)ppGpp produced as a result of leaky expression of YwaC could also reduce the intracellular level of GTP by inhibiting the IMP dehydrogenase (Lopez et al. 1981). Therefore, it is possible that ppGp/pGpp helps to reduce the GTP levels more effectively than ppGpp. Another possibility is that these nucleotides inhibit some unknown enzymes that are essential for the cell growth. In any case, since the growth arrest, initiated by inducing the expression of *ywaC* gene, was only observed in cells that carried the Δ*relA* mutation, the growth-inhibitory effects of ppGp/pGpp, as well as that of (p)ppGpp, would not take place when RelA functions in the cell as the metal-dependent phosphohydrolase.

We similarly used CE-TOFMS to determine the effects of *yjbM* gene expression on the intracellular concentrations of various guanine nucleotides in the Δ*relA* Δ*yjbM* Δ*ywaC* triple mutant. As shown in Figure S5, intracellular GTP level was reduced, and ppGp/pGpp as well as ppGpp were detected in the YjbM-expressing RIK1059 cells. At present we do not have any clear experimental data that would suggest that the intracellular expression levels of YjbM and YwaC in the Δ*relA* Δ*yjbM* Δ*ywaC* triple mutant strain were almost same. Therefore, it was difficult to compare the activities of YjbM and YwaC with respect to the intracellular concentration of various guanine nucleotides. However, given that ppGp/pGpp as well as ppGpp were found in both IPTG-induced RIK1059 and RIK1066 cells (Figs. 3B and S5), there ought to be some critical intracellular threshold levels of these nucleotides for them to inhibit the cell growth and efficiently reduce the intracellular levels of GTP as well.

In addition to causing growth arrest, inducing the expression of *ywaC* resulted in the formation of 70S dimers in the triple mutant (Figs. 5 and 6). Results presented here also indicate that YvyD is essential for the 70S dimer formation (Figs. 6 and 7). Although it has been shown that the expression of *yvyD* is enhanced by various stress conditions such as heat, salt, and ethanol stress, as well as nutrient starvation (Drzewiecki et al. 1998), the detailed biological function of YvyD remains unclear. Results described in this study provide the first experimental evidence that YvyD is required for the formation of the 70S dimer in *B. subtilis*.

In *E. coli*, two proteins, RMF and HPF (hibernation promoting factor) are required for the formation of 100S ribosomes (Wada et al. 1990; Ueta et al. 2005, 2008). The initial dimerization of 70S ribosomes is dependent on RMF, resulting in the formation of immature 90S ribosomes. Subsequently, 100S ribosomes are generated from these immature ribosomes by binding to both RMF and HPF (Ueta et al. 2005, 2008). The 100S ribosome is translationally inactive and is found in the *E. coli* cells only during the stationary phase (Wada et al. 1990, 1995, 2000), which suggests that ribosomes enter into hibernation stage for survival during the stationary phase. Orthologues of the *rmf* gene are, however, found only in γ-proteobacteria, which suggests that other types of bacteria use different mechanisms to inactivate ribosome by forming 70S dimers (Ueta et al. 2008). On the other hand, genes encoding the homologues of HPF protein are found in many bacteria and plants (Ueta et al. 2008). A computer-aided search showed that YvyD shares 38% identity with and 51% similarity to the amino acid sequence of HPF. However, the YvyD protein also contained additional 88 amino acids in its C-terminus end. These results suggest that YvyD is a member of the long HPF family that is distributed widely among bacteria (except for γ-proteobacteria) and plants (Ueta et al. 2008). Therefore, results presented here strongly suggest that a protein that belonged to the long HPF family (i.e., YvyD) is required for the 70S dimer formation in *B. subtilis*. Recently, it was shown that the hibernation promoting factor homolog, SaHPF, of *Staphylococcus aureus* was able to convert 70S ribosomes into 100S ribosomes in vitro by itself (Ueta et al. 2010). Therefore, the mode of action of this protein might differ from the modes of action of HPF and RMF that are involved in the 70S dimer formation in *E. coli*. We, however, found that efficient dimerization of 70S ribosomes was not observed when YvyD was overexpressed in the wild-type cells during exponential growth phase of *B. subtilis* (Fig. 9B), which suggested that YvyD does not directly trigger the formation of 70S dimers in these cells. The disparity between these results from those of the previous studies (Ueta et al. 2010) might be due to the activity of ribosome itself. In the exponentially growing cells, most of ribosomes are thought to be active as the translational apparatus. On the other hand, high-salt washed 70S ribosomes used for the in vitro 100S formation assay using the purified SaHRP protein (Ueta et al. 2010) did not act as the translational apparatus in the reaction mixture, even though their activity might have remained unchanged. Therefore, it is most likely that YvyD, as well as SaHPF, is targeted only to the inert form of 70S ribosomes. Given that induction of YwaC expression in the Δ*relA* Δ*yjbM* Δ*ywaC* triple mutant caused growth arrest, which was independent of the presence or absence of the *yvyD* gene (Figs. 2, 5, and 7), it seems likely that the inert form of 70S ribosome is generated rapidly at first by the induction of YwaC expression in the Δ*relA* Δ*yjbM* Δ*ywaC* triple mutant, which in part triggers the growth arrest and then these ribosomes are converted into 70S ribosome dimers by the expressed YvyD. Further in vitro studies using purified YvyD and 70S ribosome are needed to clarify the mechanism by which the 70S dimer is formed in *B. subtilis*.

The active transcription of *yvyD* gene following the induction of the *ywaC* gene in the triple null mutant was shown to depend on σ^H^ (Fig. 8), a sporulation-specific sigma factor that is involved in the transcription of early sporulation genes (Chibazakura et al. 1991; Healy et al. 1991; Haldenwang 1995). As we were unable to find 70S dimers following the IPTG-induced expression of YwaC in the wild-type RIK1052 cells (Fig. S3), it is likely that the expression of YwaC does not induce the transcription of *yvyD* directly. It has been reported earlier that amino acid starvation activates σ^H^ and that this activation depends in part on the function of RelA (Eymann et al. 2001, 2002), which suggests that appreciable levels of (p)ppGpp are required for the activation of σ^H^. However, the underlying mechanism for the activation of σ^H^ by (p)ppGpp remains unknown. In this study, we have shown that besides the expression of the *yvyD* gene expressions of several other σ^H^-dependent genes (such as *spoVG*, *spo0A*, and *kinA* and *ytxGHJ*) were indeed increased (Fig. 4; Tables S2 and S3). On the other hand, expressions of various genes encoding ribosomal proteins, such as the genes found in the S10-spc cluster, were decreased in the IPTG-induced RIK1066 cells that were expressing YwaC (Fig. 4A; Tables S2 and S3). It has been shown that transcription from the *B. subtilis rrnB* P1 promoter is not inhibited by the addition of ppGpp in vitro (Krásný and Gourse 2004), indicating that ppGpp cannot directly inhibit the transcription from stringently controlled promoters, as was the case in *E. coli* (Paul et al. 2004). Instead, it was proposed that the reduction in the intracellular GTP level in response to amino acid starvation causes downregulation of stringently controlled promoters with +1G start sites (Krásný et al. 2008; Natori et al. 2009; Tojo et al. 2010). The transcriptional start sites in *spoVG* and *yvyD* are T or A (*spoVG*; Moran et al. 1981) and A (*yvyD*-Pσ^H^; Drzewiecki et al. 1998 and Fig. 8B), respectively. The S10-spc gene cluster was transcribed from two σ^A^-dependent promoters, both of which begin with a G (Li et al. 1997). Since the amount of (p)ppGpp produced in the IPTG-induced RIK1066 cells was regarded as the basal level, it would exclude the possibility that (p)ppGpp directly regulates the activity of RNA polymerase. Instead, we believe that severe reduction in the GTP level caused by (p)ppGpp or ppGp/pGpp was rather critical for the observed changes in the transcriptional profiles. Under this situation, the stringently controlled promoters with +1G start sites would be downregulated, whereas the promoters with +1A start sites would be upregulated. Given that all the rRNA encoding genes have stringently controlled promoters with +1G start sites (Natori et al. 2009), there would be reduced transcription of the rRNA genes. Therefore, many of the RNA polymerase core subunits would be virtually free from σ^A^ protein. Consequently, the σ^H^ protein would be able to easily access the RNA polymerase core enzymes and would then transcribe promoters with +1A start sites, even though cells are in the exponential growth phase. Further study will be needed to clarify this hypothesis.

**Figure 10:**
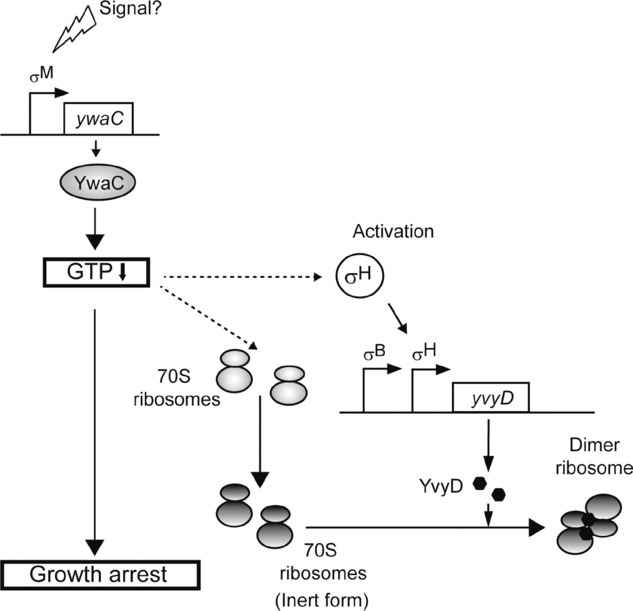
Model depicting the dimerization mechanism of the 70S ribosome of *Bacillus subtilis* (see text).

From the results obtained in this study, we have proposed a model (shown in Fig. 10) illustrating the function of YwaC as well as the formation of the 70S dimer. In this model, the transcription of *ywaC* is dependent on σ^M^, an extracytoplasmic function (ECF) sigma factor that is activated by various stress conditions including high salinity, ethanol, heat, acid stresses, and exposure to several antibiotics that affect the cell wall (Eiamphungporn and Helmann 2008). Therefore, *ywaC* is actively transcribed by RNA polymerase containing the activated σ^M^ when adverse environmental conditions are sensed by σ^M^. The expressed YwaC causes a decrease in the intracellular GTP level as well as synthesize various guanine nucleotides only when RelA is inactive or not expressed in the cells. The reduction in the GTP level triggers the activation of σ^H^ and then the RNA polymerase containing σ^H^ transcribes the *yvyD* gene. Under this situation, many ribosomes are not able to actively translate various proteins due to the reduction in the GTP level or presence of various guanine nucleotides. The expressed YvyD protein is then able to target the inert form of 70S ribosomes in the cells and promotes dimerization of the 70S ribosomes. The 70S dimers formed in *B. subtilis* cells would be translationally inactive as in *E. coli*. As a result, the transcription of *ywaC* as well as the intracellular level of YwaC protein in the wild-type cells are weakly induced during the entry into the stationary phase (Nanamiya et al. 2008) and activation of σ^H^ is required for the initiation of sporulation (Healy et al. 1991; Haldenwang 1995). This model would account for the inactivation of ribosomes (in the stationary phase) to allow cell survival during the stationary phase and would also allow sporulation. In fact, the intracellular level of YvyD protein was increased after the end of the exponential growth phase (Fig. 9C), and YvyD protein was found in the ribosome fractions prepared from the cells harvested during the stationary phase (Nanamiya et al. 2004). Given that large amounts of GTP are consumed during the translation, inactivation of ribosome is likely to be one of the most important mechanisms for cell survival when the intracellular GTP levels are drastically reduced. It is, however, not clear at this point whether the regulatory mechanism contributing to this inactivation process was due to the expressed YwaC, which functions as the synthetase of various guanine nucleotides, or due to the absence of RelA as the hydrolase against various guanine nucleotides that are synthesized by YwaC. It also remains unclear how RelA is regulated during the stationary phase as well as during sporulation. Thus, elucidation of the mechanisms by which YwaC and YvyD are involved in the inactivation and preservation of the ribosomes during the stationary phase, as well as during the sporulation phase, would certainly be a very interesting subject for further study.

## Experimental Procedures

### Strains

All *B. subtilis* strains used in this study were isogenic with *B. subtilis* strain 168, and are listed in Table 1. All oligonucleotide primers used in this study are listed in Table S4. Strain RIK1004 (*trpC2 ywaC*::*cat*) was constructed as follows. Oligonucleotide primers (Table S4) were used to amplify the upstream (YwaCUF and YwaCUR) and downstream (YwaCDF and YwaCDR) regions of *ywaC*. Next, the chloramphenicol resistance gene of pCBB31 (Imamura et al. 2004) was amplified by polymerase chain reaction (PCR) using primers CAT-F and CAT-R. The three fragments obtained (i.e., the upstream and downstream regions of *ywaC* and the chloramphenicol resistance gene) were used simultaneously as the template for PCR amplification with primers YwaCUF and YwaCDR. The resulting product was transformed into *B. subtilis* 168 and chloramphenicol-resistant transformants were selected on LB plates. Correct integration of *ywaC* and *cat* genes was confirmed by PCR and DNA sequence analyses. Strain RIK1051 (*trpC aprE*::P*spac-yjbM spc*) was constructed as follows. A *Sal*I-*Eco*RI-digested PCR fragment that was amplified from chromosomal DNA of 168 strain by using the primers YjbMPF (*Sal*I site underlined) and YjbMPR (*Eco*RI site underlined) was cloned into pAPNC213 (Morimoto et al. 2002). The resulting recombinant plasmid, which contained the *yjbM* gene under the control of an IPTG-inducible P*spac* promoter, was linearized with *Sca*I. It was then transformed into *B. subtilis* 168 and integrated into the *aprE* site of the chromosome by double crossing over and selection for resistance to spectinomycin. Correct integration was verified by PCR and DNA sequence analyses. Strain RIK1052 (*trpC aprE*::P*spac-yjbM spc*) was constructed in the same manner as the strain RIK1051 by using primers YwaCPF and YwaCPR. To obtain a transcriptional fusion between the *rrnO* promoter and the kanamycin-resistance gene (P*_rrnO_kan)*, a strain RIK1420, in which a kanamycin-resistance gene that lacked a promoter or a Rho-independent terminator sequence was integrated downstream of the *rrnO* P2 promoter (*rrnO2*+::*kmpt1*), was constructed as follows. First, oligonucleotide primers were used to amplify the *rrnO* promoter (primers rrnO-catF1 and rrnO-kanR1) and the 16S rRNA gene (primers rrnA-kanF2 and rrnX-catR2). Next, the *kan* gene of pUB110 was amplified with primers kanSDMF and kanR. The primer kanSDMF was designed to modify the Shine-Dalgarno sequence of the *kan* gene (GGGAA → GGAGG). The three PCR products (i.e., the *rrnO* promoter, 16S rRNA gene, and *kan* gene) were mixed and a portion of the mixture was subjected to PCR amplification using the primers rrnO-catF1 and rrnX-catR2. The resulting DNA product was used to transform *B. subtilis* strain 168, and kanamycin-resistant transformants were selected. Correct integration was confirmed by PCR and DNA sequence analyses. The synthesized P*_rrnO_kan* gene of RIK1420 was used to construct a disrupted form of the *yvyD* as follows. First, oligonucleotide primers were used to amplify the upstream (primers YvyDUF and YvyDUR) and downstream (primers YvyDDF and YvyDDR) regions of *yvyD*. Next, the P*_rrnO_kan* gene of RIK1420 was amplified by PCR using the primers rrnOFout and kanR. The products of these three PCR amplifications were used simultaneously as the template for a second PCR amplification using the primers YvyDUF and YvyDDR. The resulting product was transformed into *B. subtilis* 168 and kanamycin-resistant transformants were selected on LB plates. Correct integration was confirmed by PCR and DNA sequence analyses, and the transformant obtained was named RIK1069 (*trpC2 yvyD*::P*rrnOkan*). Strain RIK1088 (*trpC2 spo0H*::*tet*) was constructed in the similar manner as the strain RIK1069 using the primer pairs SigHUF and SigHUR, SigHDF and SigHDR, and Tet-F and Tet-R to amplify the upstream and downstream regions of *spo0H* and the tetracycline resistance gene (*tet*) of pBEST307 (Itaya 1992), respectively.

**Table 1:** *Bacillus subtilis* strains used in this study

Strain	Genotype	Source or references
168	*trpC2*	Laboratory stock
RIK900	*trpC2 relA*::*erm*	Nanamiya et al. (2008)
RIK1000	*trpC2* Δ*yjbM*	Nanamiya et al. (2008)
RIK1003	*trpC2*Δ*yjbM ywaC*::*spc relA*::*erm*	Nanamiya et al. (2008)
RIK1004	*trpC2 ywaC*::*cat*	This study
RIK1051	*trpC aprE*::P*spac-yjbM spc*	This study
RIK1052	*trpC aprE*::P*spac-ywaC spc*	This study
RIK1054	*trpC relA*::*erm aprE*::P*spac-ywaC spc*	RIK900 → *RIK1052
RIK1055	*trpC* Δ*yjbM aprE*::P*spac-yjbM spc*	RIK1051 → *RIK1000
RIK1056	*trpC* Δ*yjbM aprE*::P*spac-waC spc*	RIK1052 → *RIK1000
RIK1057	*trpC* Δ*yjbM ywaC*::*cat aprE*::P*spac-yjbM spc*	RIK1004 → *RIK1055
RIK1058	*trpC* Δ*yjbM ywaC*::*cat aprE*::P*spac-yjbM spc*	RIK1004 → *RIK1056
RIK1059	*trpC2* Δ*yjbM ywaC*::*cat relA*::*erm aprE*::P*spac-yjbM spc*	RIK900 → *RIK1057
RIK1066	*trpC2* Δ*yjbM ywaC*::*cat relA*::*erm aprE*::P*spac-ywaC spc*	RIK900 → *RIK1058
RIK1068	*trpC2 relA*::*erm* Δ*yjbM aprE*::P*spac-ywaC spc*	This study
RIK1069	*trpC2 yvyD*::P*rrnOkan*	This study
RIK1070	*trpC2* Δ*yjbM ywaC*::*cat relA*::*erm aprE*::P*spac-ywaC spc yvyD*::P*rrnOkan*	RIK1069 → *RIK1066
RIK1088	*trpC2 spo0H*::*tet*	This study
RIK1089	*trpC2* Δ*yjbM ywaC*::*cat relA*::*erm aprE*::P*spac-ywaC spc spo0H*::*tet*	RIK1088 → *RIK1066
RIK1096	*trpC2 ywaC*::*cat aprE*::P*spac-yjbM spc*	RIK1004 → *RIK1052
RIK1098	*trpC2 relA*::*erm ywaC*::*cat aprE*::P*spac-ywaC spc*	This study
RIK1371	*trpC2* Δ*yjbM ywaC*::*cat relA*::*erm aprE*::P*spac-ywaC L176F spc*	This study
RIK1375	*trpC2* Δ*yjbM ywaC*::*cat relA*::*erm aprE*::P*spac-ywaC D87G spc*	This study
RIK1392	*trpC2* pULI7*yvyD*	This study
RIK1420	*trpC2 rrnO2*+::*kmpt1*	This study

* Transformation.

pULI7yvyD, carrying IPTG inducible *yvyD* gene, was constructed as follows. A XbaI-digested PCR fragment that was amplified from chromosomal DNA of 168 strain by using the primers yvyD-xbaIF (XbaI site underlined) and yvyD-xbaIR (XbaI site underlined) was cloned into pULI7 (Ashikaga et al. 2000). Proper insertion of *yvyD* into pULI7, which formed a transcriptional fusion between the IPTG-inducible P*spac* promoter and *yvyD* gene, was confirmed by DNA sequencing.

The other strains used in this study were obtained by transformation as shown in Table 1.

### Media

The media used included LB and LB agar (Sambrook and Russell 2001). When required, antibiotics were added at the following concentrations: chloramphenicol 5 μg mL^−1^, erythromycin 0.5 μg mL^−1^, spectinomycin 100 μg mL^−1^, kanamycin 5 μg mL^−1^, and tetracycline 10 μg mL^−1^.

### Isolation of the suppressor mutants that can bypass the growth inhibition caused by the induction of the P*spac-ywaC* gene in the Δ*relA* Δ*yjbM* Δ*ywaC* mutant

RIK1066 (*aprE*::P*spac*-*ywaC* Δ*relA* Δ*yjbM* Δ*ywaC*) mutant cells were grown in LB medium overnight at 37°C, plated on LB agar medium containing 100 μg mL^−1^ of spectinomycin and 1 mM IPTG, and incubated overnight at 37°C. Appeared colonies were purified by single-colony isolation technique, and their growth characteristics were examined and nature of their mutations was determined.

### Western blot analyses

Crude cell extracts containing 20 μg of total proteins were electrophoresed on sodium dodecyl sulfate—12% polyacrylamide minigel and the separated proteins were transferred to a polyvinilidene difluoride (PVDF) membrane (Millipore Co., Japan). Immunodetection procedures were carried out as described previously (Nanamiya et al. 2003). Anti-YjbM, anti-YwaC, and anti-YvyD antibodies were prepared by immunizing rabbits with the synthetic oligo-peptides (C-IEHSLNYKYSGNIP and C-EMSEIRGEVQEAQA for YjbM, C-IEHTKSRVKSFESI and C-VPKQLTDELKEAAE for YwaC, and C-KFYNDKESKVEVTI and C-NKLERQIRKHKTKV for YvyD; the first cysteine residue is added synthetically in order to conjugate the oligo-peptide to Keyhole Limpet Hemocyanin [KLH] protein) corresponding to the amino acid positions 153–166 and 186–199 of YjbM, positions 52–65 and 180–193 of YwaC, and positions 44–57 and 82–95 of YvyD, respectively. The antibodies were used at a dilution of 1:1000 (for anti-YjbM- and anti-YvyD antibodies) or at a dilution of 1:10,000 (for anti-YwaC antibody).

### Determination of metabolites

*Bacillus subtilis* RIK1059 (*aprE*::P*spac*-*yjbM* Δ*relA* Δ*yjbM* Δ*ywaC*) and RIK1066 (*aprE*::P*spac*-*ywaC* Δ*relA* Δ*yjbM* Δ*ywaC*) were pregrown separately in LB agar plates containing 5 μg/mL of chloramphenicol, 0.5 μg/mL of erythromycin, and 100 μg/mL of spectinomycin at 28°C for 12 h. Several fresh colonies appearing on each plate were inoculated into LB liquid medium and incubated at 37°C with shaking. When the cells reached the middle logarithmic phase (optical density at 600 nm = 0.5), the culture was supplemented with 1 mM IPTG and was continuously shaken at 37°C until the sampling times. Intracellular metabolites were extracted as described previously (Ohashi et al. 2008). Cells grown as described above were collected for metabolite analysis at indicated times. An aliquot of the cell culture containing approximately 10^9^ cells was filtered by vacuum filtration system using a 0.4-μm pore size filter. Cells remaining on the filter were washed twice with Milli-Q water. The filter was immersed in 2 mL of methanol including 5 μM each of internal standards, methionine sulfone and D-camphor-10-sulfonic acid (CSA). The dish was sonicated for 30 sec using an Elma Transsonic T460/H ultrasonic syringe (Elma Hans Schmidbauer GmbH & Co., Singen, Germany) to resuspend the cells completely. A 1.6 mL portion of the methanol cell suspension was transferred to a 15-mL Falcon Blue Max Jr., 352097 centrifuge tube (Becton Dickinson & Co., Franklin Lakes, NJ), and mixed with 1.6 mL of chloroform and 640 μL of Milli-Q water. After vortexing vigorously, the mixture was centrifuged at 4600 *g* for 5 min at 4°C. The aqueous layer (750 μL) was carefully transferred to an Amicon Ultrafree-MC ultrafilter tip (Millipore Co.) and then centrifuged at 9100 *g* for approximately 2 h at 4°C. The filtrate was dried and preserved at −80°C until used for further analysis. Prior to analysis, the sample was dissolved in 25 μL of Milli-Q water. CE-TOFMS was carried out using an Agilent Capillary Electrophoresis System equipped with an Agilent 6210 Time of Flight mass spectrometer, Agilent 1100 isocratic HPLC pump, Agilent G1603A CE-MS adapter kit, and Agilent G1607A CE-ESI-MS sprayer kit (Agilent Technologies, Waldbronn, Germany). The system was controlled by the Agilent G2201AA ChemStation software version B.03.01 for CE (Agilent Technologies). Data acquisition was performed using the Analyst QS Build: 7222 software for Agilent TOF (Applied Biosystems, Foster City, CA/ MDS Sciex, Ontario, Canada). The extracted metabolites were analyzed by CE-TOFMS as described previously (Ooga et al. 2011) using a commercial electrophoresis buffer (Solution ID H3302–1021; Human Metabolome Technologies Inc., Tsuruoka, Japan). Peak extraction was carried out with the open source software MathDAMP 28 with modifications. The peak alignment was performed according to the *m*/*z* value and normalized migration time. Subsequently, peak areas were normalized against those of the internal CSA standards for anionic metabolites. The resultant relative areas were further normalized by sample amount (OD values).

### High-density transcriptome analysis

The custom Affymetrix tiling chip used for this analysis contained 55,430 probes (each 25 mer) for the coding strand of the protein-coding regions at 25–30 bp intervals and 72,218 probes for both strands of the intergenic regions at 2–3 bp intervals. Total RNAs were extracted from *B. subtilis* cells (10 OD_600_) as described previously (Ishikawa et al. 2007). Synthesis of cDNA, terminal labeling, and oligonucleotide chip hybridization were performed according to the supplier's instruction manual (Affymetrix, Cleveland, OH). Briefly, cDNA was synthesized from 10 μg of total RNA using random primers and reverse transcriptase (Superscript III, Invitrogen), followed by purification of cDNA using QiaQuick columns (Qiagen) and digestion with DNase I (GE Healthcare Bioscience). Next, cDNA fragments were terminally labeled with biotin-ddUTP using an ENZO BioArray Terminal Labeling Kit (Enzo Life Sciences). Hybridization with the oligonucleotide chip was performed for 16 h at 42°C, followed by washing, staining, and scanning using the GeneChip Instrument System according to the manufacturer's instructions (Affymetrix). Transcriptional signals were analyzed and visualized along the genome coordinate with the in silico Molecular Cloning program, Array Edition (*In Silico* Biology). For each experiment, the signal intensities were adjusted to confer a signal average of 500. The average signal intensities of probes in each coding sequence were calculated after the removal of the lowest and highest intensities.

### Sucrose density gradient sedimentation analysis

Cells were grown in LB medium at 37°C with shaking and were harvested at the indicated times. Cells were resuspended in Buffer I (20 mM Tris-HCl pH 7.6, 10 mM magnesium acetate, 100 mM ammonium acetate, 6 mM β-mercaptoethanol, and 2 mM phenylmethylsulfonylfluoride) and then disrupted by passing through a French pressure cell (Aminco) at 8000 psi. After removal of cell debris by centrifugation at 12,000 *g* for 30 min at 4°C, aliquots of the supernatant (10 A_260_ units per tube) were layered onto 10–40% sucrose density gradients and centrifuged at 65,000 *g* for 17.5 h at 4°C (Hitachi P40ST rotor). Absorbance profiles were monitored at 254 nm using a Piston Gradient Fractionator (Bio ComP) and Bio-mini UV Monitor (ATTO).

### Recovery of 70S and additional peak fractions

Aliquots of crude cell extract, prepared as described above, were centrifuged at 12,000 *g* for 30 min at 4°C to remove cell debris. The supernatants obtained were centrifuged further at 30,000 *g* for 30 min at 4°C in a Hitachi P55ST2 rotor and then the resulting supernatants were centrifuged at 9500 *g* for 90 min at 4°C in a Hitachi P55ST2 rotor. The pellets were resuspended in 100 μL of Buffer I and subjected to centrifugation through a 10–40% sucrose density gradient (Hitachi P28S rotor, 100 A_260_ units per tube) at 40,000 *g* for 16 h. Absorbance profiles were monitored at 254 nm using a Piston Gradient Fractionator (Bio ComP) and Bio-mini UV Monitor (ATTO), and the fractions corresponding to the 70S peak and the additional peak were collected and pooled. The pooled fractions were diluted with Buffer I (1:2 dilutions), and the ribosomes were recovered by centrifugation in a Hitachi P55ST2 rotor at 230,000 *g* for 3 h at 4°C. The resulting ribosomal pellets were resuspended in 100 μL of Buffer I and then stored at −80°C until use. RFHR 2D polyacryamide gel electrophoresis (PAGE) (Wada 1986) was carried out essentially as described previously (Nanamiya et al. 2004, 2006).

### Negative stain electron microscopy

For the negative staining, ribosome samples were prepared by centrifugation as described above and suspended in Buffer I. Polyvinyl formal-coated 200 mesh nickel grid was placed on a drop of ribosomal suspension and the extra liquid on the grid was then removed by touching the edge to a piece of filter paper. Afterward, the grid was stained for 10 sec on a drop of 3% uranyl acetate. The specimens were examined with a JEM-1230 electron microscope (JEOL).

### Peptide mass fingerprinting

Stained spots were excised manually from the 2D-PAGE gels. The gel slices were destained and digested with sequencing-grade modified trypsin (Promega) as described previously (Katayama et al. 2001), with minor modifications. After digestion, tryptic peptide fragments were extracted from the gel pieces with 5% (v/v) formic acid and 50% (v/v) acetonitrile. Extracts were concentrated to a volume of less than 5 μL in a MicroVac MV-100 vacuum centrifuge (TOMY Digital Biology). The concentrated peptides were desalted using ZipTip μ-C18 pipette tips (Millipore) and spotted into the wells of a 384-well plate (Kratos Analytical) for further processing. After the samples were dried, 0.5% (w/v) α-cyano-4-hydroxycinnamic acid in 50% (v/v) acetonitrile and 0.1% (v/v) trifluoroacetic acid was spotted on the peptide spots and allowed to dry. The samples were subsequently analyzed by PMF (peptide mass fingerprinting) and PSD-MS/MS (post source decay-tandem mass spectrometry) ion search using an AXIMA-TOF^2^ mass spectrometer (Shimadzu) in reflectron mode. Database searches were performed using the software program MASCOT v2.2.01 (Matrix Science) and a local database, which was built from the FASTA file of 4106 open reading frames of *B. subtilis* obtained from the SubtiList Web Server (http://genolist.pasteur.fr/SubtiList/). As search parameters, carbamidomethylation of cysteine residues was designated as a fixed modification, and methionine oxidation was designated as a variable modification. The permissible value of missed cleavages was set to one. MS tolerance values were set at 0.2–0.3 Da (peptide mass tolerance of PSD) or 0.8 Da (fragment mass tolerance of MS/MS). For each sample, the MS analyzer was calibrated by an internal calibration method with trypsin autolysis peaks (*m*/*z* = 842.51 and 2211.10).

### Primer extension analysis

Cells were grown in LB medium at 37°C with shaking and were harvested at the times indicated. Total RNA (30 μg) extracted from the cells and 1 pmol of IRD (infrared dye) dye-labeled oligonucleotide primer yvyDPE.r (5′-GCGTTGTACATATCCTCGTTATGCACCTCG-3′), which is complementary to the nucleotide sequence of the 5′-terminal region of the *yvyD* gene, were mixed and reverse transcription was carried out using SuperScript III reverse transcriptase (Invitrogen) as described previously (Nanamiya et al. 2003, 2004; Natori et al. 2007). The products were separated on 5% polyacrylamide per 6 M urea gels alongside a sequencing ladder generated by PCR cycle sequencing with yvyDPE.r, which facilitated mapping of the 3′ ends of the reverse transcripts (i.e., the 5′ ends of the RNAs). The template for the sequencing ladder was synthesized by PCR using primers YvyDTF (the underlined sequence of the primer shown in Table S4 represents the recognition site for the M13 forward primer) and YvyDTR. IRD-labeled reverse transcription and sequencing products were detected by using a LI-COR model 4300 DNA Analyzer (ALOKA).

### Northern blot analysis

Total RNA (15 μg) was subjected to Northern blot analysis as described (Nanamiya et al. 2003). The digoxigenin-labeled *yvyD* probe was synthesized with a DIG RNA labeling kit (Roche Diagnostics). The template for *yvyD* probe was amplified from *B. subtilis* strain 168 by PCR using the primers YvyD-PF and YvyD-PR (the underlined sequences shown in Table S4 represent a T7 RNA polymerase recognition site). ECF substrate (GE Healthcare), which is a fluorescent substrate for alkaline phosphatase, was used to amplify the signal and the amplified signal was detected using a Typhoon 9210 Variable Mode Imager (GE Healthcare).

## Supporting Information

**Figure S1.** Growth profiles of various strains harboring P*spac*-*ywaC* or its variants, P*spac*-*ywaCL176F* or P*spac*-*ywaCD87G,* in LB medium.

**Figure S2.** Clustering analysis of genes affected by the induction of the transcription of *ywaC*.

**Figure S3.** Sucrose density gradient sedimentation analysis.

**Figure S4.** Western blot analysis of YwaC.

**Figure S5.** Intracellular concentration of (**A**) GTP, (**B**) ppGp/pGpp, and (**C**) ppGpp in cells: (○) RIK1059 (*aprE*::P*spac*-*yjbM relA*::*erm* Δ*yjbM ywaC*::*cat*) and (?) RIK1066 (*aprE*::P*spac*-*ywaC relA*::*erm* Δ*yjbM ywaC*::*cat*).

**Table S1.** Location and identification of mutation in the *Pspac-ywaC* gene.

**Table S2.** Whole data set of transcription levels obtained in this study.

**Table S3.** Cluster analysis of the transcription of the genes affected by the expression of *ywaC* in *relA yjbM ywaC* triple mutant.

**Table S4.** Oligonucleotides used in this study.

## References

[b1] Aravind L, Koonin EV (1998). The HD domain defines a new superfamily of metal-dependent phosphohydrolases. Trends Biochem. Sci.

[b2] Artsimovitch I, Patlan V, Sekine S, Vassylyeva MN, Hosaka T, Ochi K, Yokoyama S, Vassylyev DG (2004). Structural basis for transcription regulation by alarmone ppGpp. Cell.

[b3] Ashikaga S, Nanamiya H, Ohashi Y, Kawamura F (2000). Natural genetic competence in *Bacillus subtilis natto* OK2. J. Bacteriol.

[b4] Battesti A, Bouveret E (2006). Acyl carrier protein/SpoT interaction, the switch linking SpoT-dependent stress response to fatty acid. Mol. Microbiol.

[b5] Cashel M, Gentry DR, Hernandez VJ, Vinella D, Neidhardt F (1996). The stringent response. Escherichia coli and Salmonella: cellular and molecular biology.

[b6] Chibazakura T, Kawamura F, Takahashi H (1991). Differential regulation of *spo0A* transcription in *Bacillus subtilis*: glucose represses promoter switching at the initiation of sporulation. J. Bacteriol.

[b7] Das B, Pal RR, Bag S, Bhadra RK (2009). Stringent response in *Vibrio cholerae*: genetic analysis of *spoT* gene function and identification of a novel (p)ppGpp synthetase gene. Mol. Microbiol.

[b8] Diesterhaft MD, Freese E (1973). Role of pyruvate carboxylase, phosphoenolpyruvate carboxykinase, and malic enzyme during growth and sporulation of *Bacillus subtilis*. J. Biol. Chem.

[b9] Drzewiecki K, Eymann C, Mittenhuber G, Hecker M (1998). The *yvyD* gene of *Bacillus subtilis* is under dual control of σ^B^ and σ^H^. J. Bacteriol.

[b10] Eiamphungporn W, Helmann JD (2008). The *Bacillus subtilis* σ^M^ regulon and its contribution to cell envelope stress responses. Mol. Microbiol.

[b11] Eymann C, Mittenhuber G, Hecker M (2001). The stringent response, σ^H^-dependent gene expression and sporulation in *Bacillus subtilis*. Mol. Gen. Genet.

[b12] Eymann C, Homuth G, Scharf C, Hecker M (2002). Bacillus subtilis functional genomics: global characterization of the stringent response by proteome and transcriptome analysis. J. Bacteriol.

[b13] Haldenwang WG (1995). The sigma factors of *Bacillus subtilis*. Microbiol. Rev.

[b14] Healy J, Weir J, Smith I, Losick R (1991). Post-transcriptional control of a sporulation regulatory gene encoding transcription factor σ^H^ in *Bacillus subtilis*. Mol. Microbiol.

[b15] Hogg T, Mechold U, Malke H, Cashel M, Hilgenfeld R (2004). Conformational antagonism between opposing active sites in a bifunctional RelA/SpoT homolog modulates (p)ppGpp metabolism during the stringent response. Cell.

[b16] Hou Z, Cashel M, Fromm HJ, Honzatko RB (1999). Effectors of the stringent response target the active site of *Escherichia coli* adenylosuccinate synthetase. J. Biol. Chem.

[b17] Imamura D, Kobayashi K, Sekiguchi J, Ogasawara N, Takeuchi M, Sato T (2004). *spoIVH**ykvV*), a requisite cortex formation gene, is expressed in both sporulating compartments of *Bacillus subtilis*. J. Bacteriol.

[b18] Inaoka T, Takahashi K, Ohnishi-Kameyama M, Yoshida M, Ochi K (2003). Guanine nucleotides guanosine 5’-diphosphate 3’-diphosphate and GTP co-operatively regulate the production of an antibiotic Bacilysin in *Bacillus subtilis*. J. Biol. Chem.

[b19] Ishikawa S, Ogura Y, Yoshimura M, Okumura H, Cho E, Kawai Y, Kuroiwa K, Oshima T, Ogasawara N (2007). Distribution of stable DnaA binding sites on the *Bacillus subtilis* genome detected using a modified ChIP-chip method. DNA Res.

[b20] Itaya M (1992). Construction of a novel tetracycline resistance gene cassette useful as a marker on the *Bacillus subtilis* chromosome. Biosci. Biotech. Biochem.

[b21] Katayama H, Nagasu T, Oda Y (2001). Improvement of in-gel digestion protocol for peptide mass fingerprinting by matrix-assisted laser desorption/ionization time-of-flight mass spectrometry. Rapid Comm. Mass. Spectrom.

[b22] Krásný L, Gourse RL (2004). An alternative strategy for bacterial ribosome synthesis: *Bacillus subtilis* rRNA transcription regulation. EMBO J.

[b23] Krásný L, Tišerová H, Jonák J, Rejman D, Šanderová H (2008). The identity of the transcription +1 position is crucial for changes in gene expression in response to amino acid starvation in *Bacillus subtilis*. Mol. Microbiol.

[b24] Kunst F, Ogasawara N, Moszer I, Albertini AM, Alloni G, Azevedo V, Bertero MG, Bessieres P, Bolotin A, Borchert S (1997). The complete genome sequence of the Gram-positive bacterium *Bacillus subtilis*. Nature.

[b25] Lemos JA, Lin VK, Nascimento MM, Abranches J, Burne RA (2007). Three gene products govern (p)ppGpp production by *Streptococcus mutans*. Mol. Microbiol.

[b26] Li X, Lindahl L, Sha Y, Zengel JM (1997). Analysis of the *Bacillus subtilis* S10 ribosomal protein gene cluster identifies two promoters that may be responsible for transcription of the entire 15-kilobase S10-spc-alpha cluster. J. Bacteriol.

[b27] Lopez JM, Dromerick A, Freese E (1981). Response of guanosine 5’-triphosphate concentration to nutritional changes and its significance for *Bacillus subtilis* sporulation. J. Bacteriol.

[b28] Magnusson LU, Farewell A, Nyström T (2005). ppGpp: a global regulator in *Escherichia coli*. Trends Microbiol.

[b29] Milon P, Tischenko E, Tomsic J, Caserta E, Folkers G, La Teana AL, Rodnina MV, Pon CL, Boelens R, Gualerzi CO (2006). The nucleotide-binding site of bacterial translation initiation factor 2 (IF2) as a metabolic sensor. Proc. Natl. Acad. Sci. U.S.A.

[b30] Mitani T, Heinze JE, Freese E (1977). Induction of sporulation in *Bacillus subtilis* by decoyinine or hadacidin. Biochem. Biophys. Res. Commun.

[b31] Mittenhuber G (2001). Comparative genomics and evolution of genes encoding bacterial (p)ppGpp synthetases/hydrolases (the Rel, RelA and SpoT proteins). J. Mol. Microbiol. Biotechnol.

[b32] Moran CP, Lang N, Banner CD, Haldenwang WG, Losick R (1981). Promoter for a developmentally regulated gene in *Bacillus subtilis*. Cell.

[b33] Morimoto T, Loh PC, Hirai T, Asai K, Kobayashi K, Moriya S, Ogasawara N (2002). Six GTP-binding proteins of the Era/Obg family are essential for cell growth in *Bacillus subtilis*. Microbiology.

[b34] Nanamiya H, Shiomi E, Ogura M, Tanaka T, Asai K, Kawamura F (2003). Involvement of ClpX protein in the post-transcriptional regulation of a competence specific transcription factor, ComK protein, of *Bacillus subtilis*. J. Boichem. (Tokyo).

[b35] Nanamiya H, Akanuma G, Natori Y, Murayama R, Kosono S, Kudo T, Kobayashi K, Ogasawara N, Park S-M, Ochi K (2004). Zinc is a key factor in controlling alternation of two types of L31 protein in the *Bacillus subtilis* ribosome. Mol. Microbiol.

[b36] Nanamiya H, Kawamura F, Kosono S (2006). Proteomic study of the *Bacillus subtilis* ribosome: finding of zinc-dependent replacement for ribosomal protein L31 paralogues. J. Gen. Appl. Microbiol.

[b37] Nanamiya H, Kasai K, Nozawa A, Yun C-S, Narisawa T, Murakami K, Natori Y, Kawamura F, Tozawa Y (2008). Identification and functional analysis of novel (p)ppGpp synthetase genes in *Bacillus subtilis*. Mol. Microbiol.

[b38] Natori Y, Nanamiya H, Akanuma G, Kosono S, Kudo T, Ochi K, Kawamura F (2007). A fail-safe system for the ribosome under zinc-limiting conditions in Bacillus subtilis. Mol. Microbiol.

[b39] Natori Y, Tagami K, Murakami K, Yoshida S, Tanigawa O, Moh Y-S, Masuda K, Wada T, Suzuki S, Nanamiya H (2009). Transcription activity of individual *rrn* operons in *Bacillus subtilis* mutants deficient of (p)ppGpp synthetase genes, *relA**yjbM* and *ywaC*. J. Bacteriol.

[b40] Ohashi Y, Hirayama A, Ishikawa T, Nakamura S, Shimizu K, Ueno Y, Tomita M, Soga T (2008). Depiction of metabolome changes in histidine-starved *Escherichia coli* by CE-TOFMS. Mol. Biosyst.

[b41] Ooga T, Sato H, Nagashima A, Sasaki K, Tomita M, Soga T, Ohashi Y (2011). Metabolomic anatomy of an animal model revealing homeostatic imbalances in dyslipidaemia. Mol. Biosyst.

[b42] Paul BJ, Barker MM, Ross W, Schneider DA, Webb C, Foster JW, Gourse RL (2004). DksA: a critical component of the transcription initiation machinery that potentiates the regulation of rRNA promoters by ppGpp and the initiating NTP. Cell.

[b43] Renna MC, Najimudin N, Winik LR, Zahler SA (1993). Regulation of the *Bacillus subtilis alsS**alsD*, and *alsR* genes involved in post-exponential phase production of acetoin. J. Bacteriol.

[b44] Sambrook J, Russell D (2001). Molecular cloning: a laboratory manual.

[b45] Seyfzadeh M, Keener J, Nomura M (1993). *spoT*-dependent accumulation of guanosine tetraphosphate in *Escherichia coli*. Proc. Natl. Acad. Sci. U.S.A.

[b46] Srivatsan A, Han Y, Peng J, Tehranchi AK, Gibbs R, Wang JD, Chen R (2008). High-precision, whole-genome sequencing of laboratory strains facilitates genetic studies. PLoS Genet.

[b47] Tojo S, Kumamoto K, Hirooka K, Fujita Y (2010). Heavy involvement of stringent transcription control depending on the adenine or guanine species of the transcription initiation site in glucose and pyruvate metabolism in *Bacillus subtilis*. J. Bacteriol.

[b48] Ueta M, Yoshida H, Wada C, Baba T, Mori H, Wada A (2005). Ribosome binding proteins YhbH and YfiA have opposite functions during 100S formation in the stationary phase of *Escherichia coli*. Genes Cells.

[b49] Ueta M, Ohniwa RL, Yoshida H, Maki Y, Wada C, Wada A (2008). Role of HPF (hibernation promoting factor) in translational activity in *Escherichia coli*. J. Biochem. (Tokyo).

[b50] Ueta M, Wada C, Wada A (2010). Formation of 100S ribosomes in *Staphylococcus aureus* by the hibernation promoting factor homolog SaHPF. Genes Cells.

[b51] Varón D, Brody MS, Price CW (1996). Bacillus subtilis operon under the dual control of the general stress transcription factor σ^B^ and the sporulation transcription factor σ^H^. Mol. Microbiol.

[b52] Vinella D, Albrecht C, Cashel M, D'Ari R (2005). Iron limitation induces SpoT-dependent accumulation of ppGpp in *Escherichia coli*. Mol. Microbiol.

[b53] Wada A (1986). Analysis of *Escherichia coli* ribosomal proteins by an improved two dimensional gel electrophoresis. I. detection of four new proteins. J. Biochem. (Tokyo).

[b54] Wada A (1998). Growth phase coupled modulation of *Escherichia coli* ribosomes. Genes Cells.

[b55] Wada A, Yamazaki Y, Fujita N, Ishihama A (1990). Structure and probable genetic location of a “ribosome modulation factor” associated with 100S ribosomes in stationary-phase *Escherichia coli* cells. Proc. Natl. Acad. Sci. U.S.A.

[b56] Wada A, Igarashi K, Yoshimura S, Aimoto S, Ishihama A (1995). Ribosome modulation factor: stationary growth phase-specific inhibitor of ribosome functions from *Escherichia coli*. Biochem. Biophys. Res. Commun.

[b57] Wada A, Mikkola R, Kurland CG, Ishihama A (2000). Growth phase-coupled changes of the ribosome profile in natural isolates and laboratory strains of *Escherichia coli*. J. Bacteriol.

[b58] Wang JD, Sanders GM, Grossman AD (2007). Nutritional control of elongation of DNA replication by (p)ppGpp. Cell.

[b59] Wendrich TM, Marahiel MA (1997). Cloning and characterization of a *relA**spoT* homologue from *Bacillus subtilis*. Mol. Microbiol.

[b60] Wendrich TM, Blaha G, Wilson DN, Marahiel MA, Nierhaus KH (2002). Dissection of the mechanism for the stringent factor RelA. Mol. Cell.

[b61] Xiao H, Kalma M, Ikehara K, Zemel S, Glaser G, Cashel M (1991). Residual guanosine 3’,5’-bisphosphate synthetic activity of *relA* null mutants can be eliminated by *spoT* null mutations. J. Biol. Chem.

